# Combined open bipolar Monteggia and Galeazzi fracture: a case report with a 1-year follow-up

**DOI:** 10.1007/s11751-017-0280-z

**Published:** 2017-03-04

**Authors:** Christos Koutserimpas, Georg Tsironis, Antonios Salasidis, Phillipp Swatoch, Konstantin Tsironis

**Affiliations:** Department of Orthopaedics Traumatology, St Katharinen, Academic Hospital of Frechen, University of Cologne, Bruesseler Platz 12, 50674 Cologne, Germany

**Keywords:** Combined Monteggia, Galeazzi, Forearm, Fracture

## Abstract

Monteggia and Galeazzi fractures account for 1–5% of total forearm fractures. A combined Monteggia and Galeazzi fracture is an extremely rare injury. We present a case of a Gustillo-Henderson type 2 open combined bipolar Monteggia and Galeazzi fracture, as well as fracture of the ulnar coronoid process in a 49-year old male. The patient was treated surgically, with open reduction and internal fixation. At 6 months postoperative, he was diagnosed with pseudarthrosis and underwent surgery with autologous bone grafting from the iliac crest. At the 1-year follow-up, the patient presented an extension deficit of 5° in elbow, a 15° deficit in pronation and 20° deficit in supination of the wrist. The patient continues to work as a painter without significant problems in his everyday routine and he is still regularly engaged in cycling. Additionally we provide a historical background of these injuries.

## Introduction

Monteggia and Galeazzi fractures are unstable injuries and account for 1–2% and 3–5% of forearm fractures, respectively [1]. A combined Monteggia and Galeazzi fracture is extremely rare. To our knowledge, only 11 cases of combined Monteggia and Galeazzi fractures are reported in the literature; two of which refer to children [2–5]. None of the reported cases refers to a bipolar Monteggia and Galeazzi injury. We report a case of a Gustillo-Henderson type 2 open combined bipolar Monteggia and Galeazzi fracture, as well as fracture of the sublime tubercle in a 49-year old male. The clinical and radiological findings, as well as the Disabilities of the Arm, Shoulder and Hand (DASH) score of the patient, during the follow-up, are also presented.

## Case presentation

**Fig. 1:**
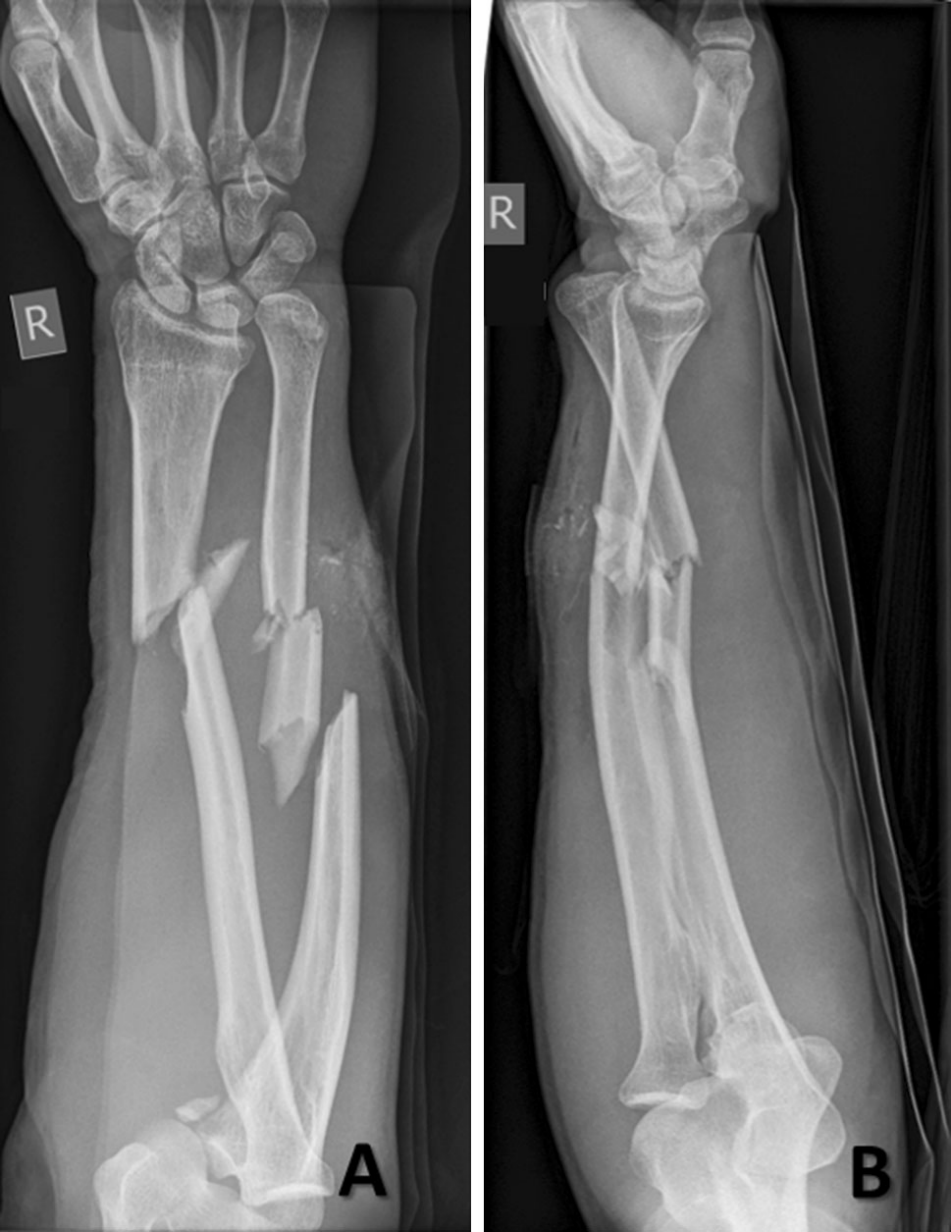
Preoperative anterior–posterior (AP) and lateral X-ray views of the forearm

**Fig. 2:**
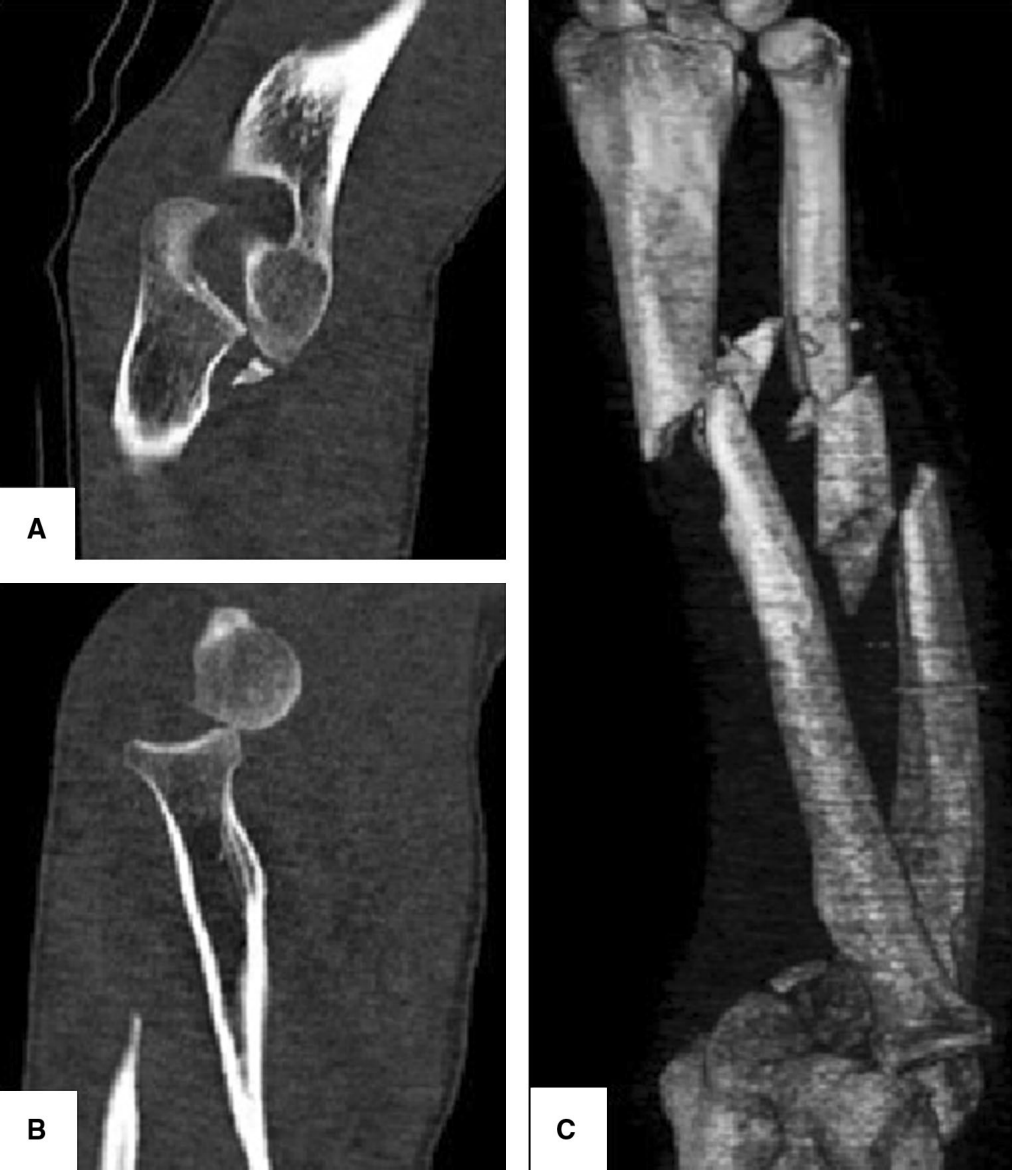
Computed Tomography sagittal views of the injured elbow (**a**, **b**) and three-dimensional (3-D) reconstruction of the described injury (**c**)

A 49-year old male was hit by a car with approximately 30 km/h, while riding a bike and fell to his right side. He was brought to our level I trauma center by an ambulance. He sustained a Gustillo-Henderson type 2 open combined bipolar Monteggia and Galeazzi fracture, as well as fracture of the sublime tubercle (Figs. 1, 2).

**Fig. 3:**
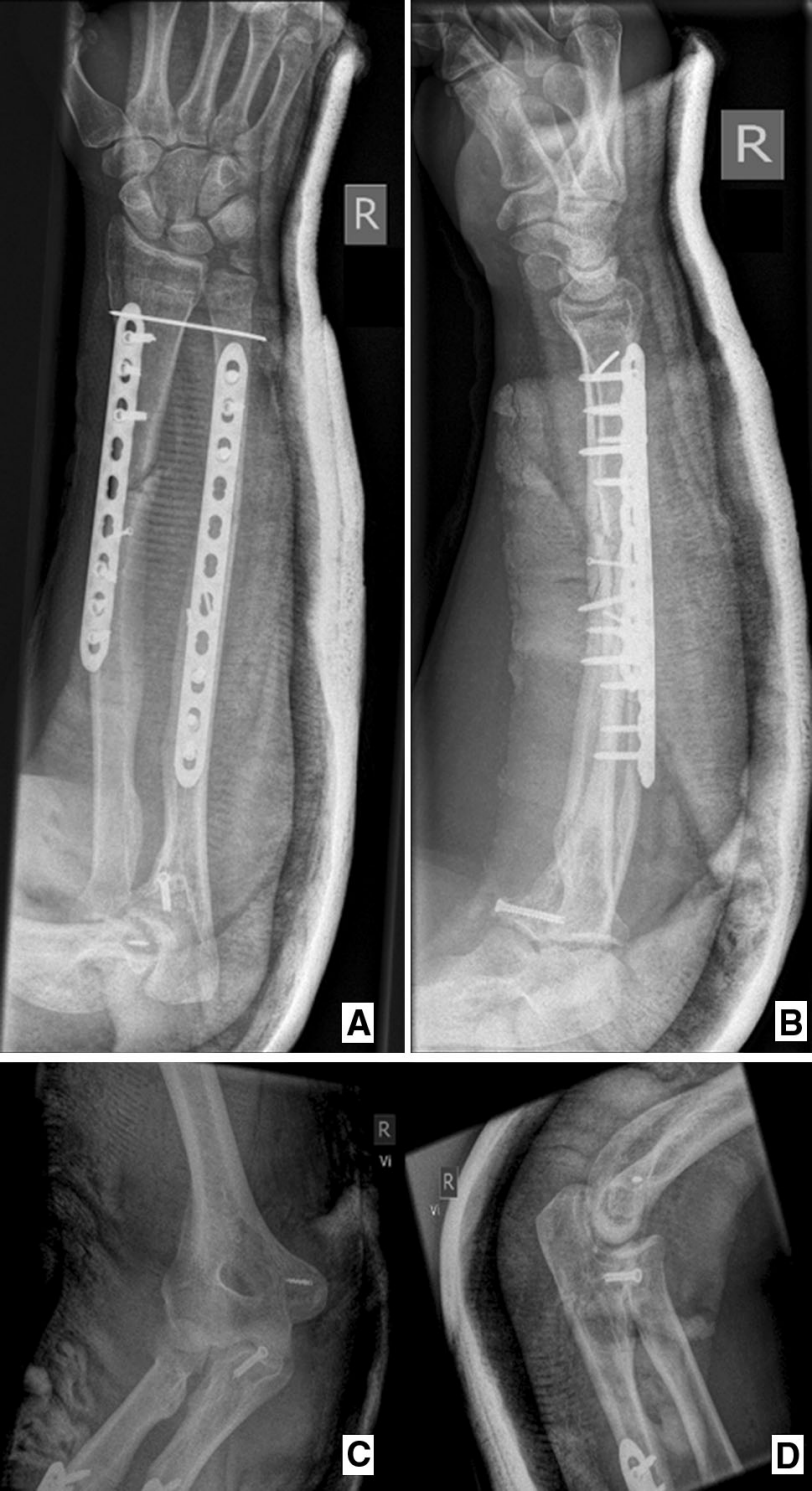
Postoperative X-rays. **a** and **b** anterior–posterior (AP) and lateral X-ray views of the forearm. **c** and **d** anterior–posterior (AP) and lateral X-ray views of the elbow

The patient underwent surgery the same day. Under general anesthesia, open reduction and internal fixation of radius and ulna were performed with the use of locking compression plates (9 and 11 holes) and 2.3-mm lag screws, while the sublime tubercle fracture was stabilized with a 3.5-mm lag screw. Furthermore, the ulnar capsular ligaments were reattached with an anchor to the medial epicondyle of the humerus. The distal radioulnar joint was stabilized with a 1.25-mm Kirschner wire. The patient’s forearm was immobilized in a long-arm cast in neutral rotation for 3 weeks (Fig. 3).

The patient received parenteral antibiotic treatment, with 1.5 g of cefuroxime per 8 h, during his hospitalization, as well as one dose at the emergency department and one while on the operating table. He made a satisfactory recovery and was discharged on the 11th postoperative day. After 1 month, the Kirschner wire in the distal radioulnar joint was removed. After cast removal, the patient was subjected to physiotherapy. The patient performed active movements, without any limits in range of motion (flexion and extension of the elbow, as well as supination and pronation). No load bearing was allowed until callus formation.

At the 4-month follow-up, the patient had active extension/flexion in the right elbow 0-5-135°, compared to 0-0-135 of the left side, while pronation/supination of the right hand was 65-0-70°, compared to 80-0-90 of the opposite healthy extremity. At the 6-month follow-up, the patient still had a 5° extension deficit in the right elbow and a 15° deficit in pronation, as well as a 20° deficit in supination in the right hand. X-rays and Computed Tomography (CT) did not show callus formation in neither the ulnar nor the radial shaft fracture; therefore, the diagnosis of atrophic pseudarthrosis was established. The patient underwent at that point surgery with autologous bone grafting from the iliac crest. After surgery, his forearm was immobilized in a posterior, above elbow cast in neutral rotation for another 4 weeks.

**Fig. 4:**
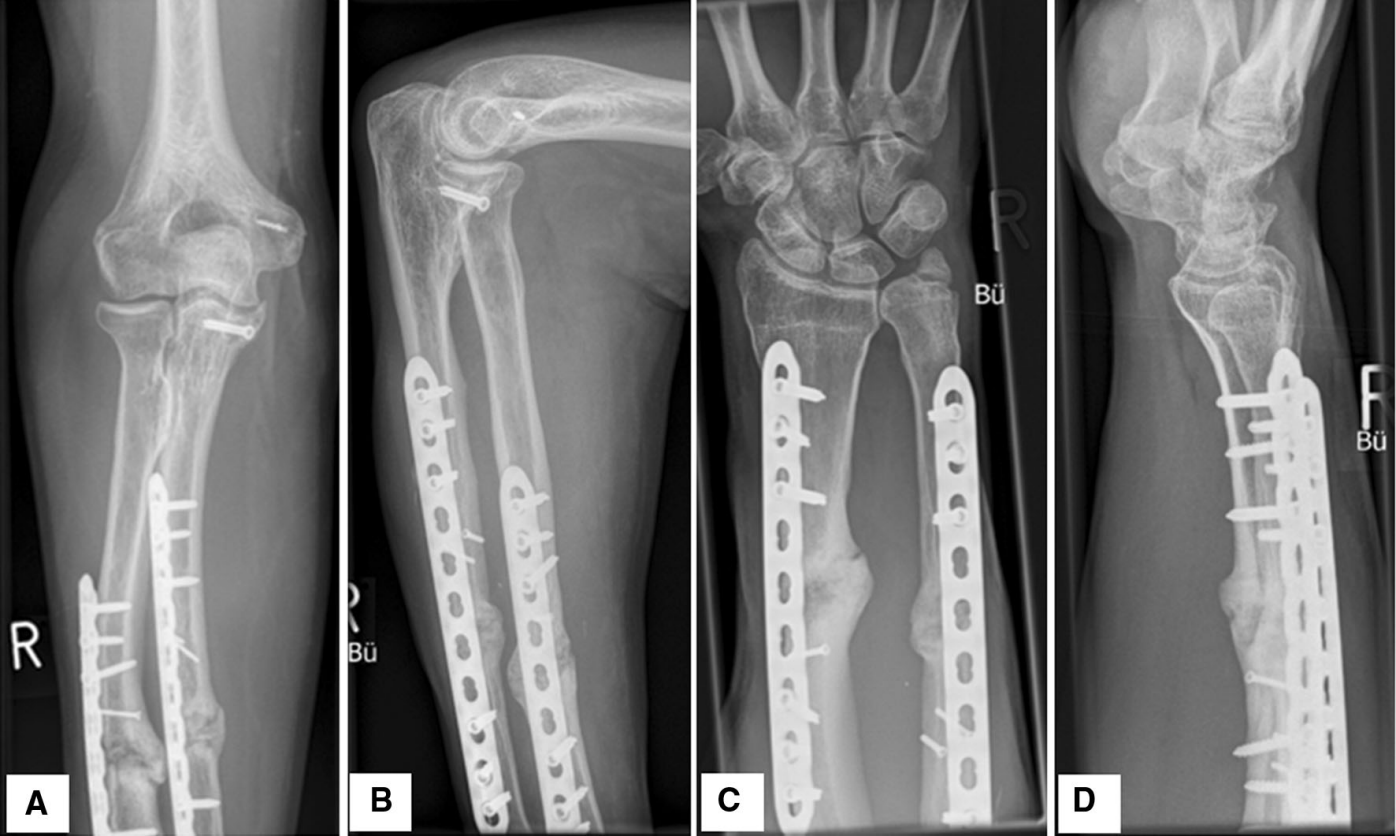
At 12-month postoperative follow-up. **a**, **b** anterior–posterior (AP) and lateral X-ray views of the right elbow. **c**, **d** anterior–posterior (AP) and lateral X-ray views of the right wrist. Sufficient callus formation is shown

At the 12-month follow-up, the patient had Disabilities of the Arm, Shoulder and Hand (DASH) score of 10. X-rays and CT showed sufficient callus formation (Fig. 4) The 5-degree deficit in elbow extension, as well as the 15-degree in hand pronation and the 20-degree one in supination remained. The extension–flexion of the right wrist was 70-0-50 compared to 80-0-60 of that of the opposite side. Radial and ulnar deviation of both wrists was 25-0-35. Although the patient is right-handed, he continues to work as a painter without significant problems in his everyday routine (DASH—work module = 6.3), and he is still regularly engaged in cycling (DASH—Sports/Performing Arts Module Score = 18.8).

## Discussion

The radial bow and the relationship between the proximal and distal radioulnar joints comprise a complex three-dimensional unit. Hence, the forearm bones are considered a single functional unit. Anatomical restoration is very important to function; therefore, any forearm shaft fracture is considered equivalent to an articular fracture [6].

The Monteggia injury was first described in 1814 by Giovanni Battista Monteggia as “radial head dislocation with ulnar fracture, located between the proximal third and base of the olecranon.” Monteggia based his findings exclusively on clinical examination and injury mechanism, since no X-rays were available at the time [7]. Later, Bado in 1967 used the term “Monteggia lesions” to classify these injuries into four types: type I, fracture of the proximal or middle third of the ulna with anterior dislocation of the radial head; type II, fracture of the proximal or middle third of the ulna with posterior dislocation of the radial head; type III, fracture of the ulnar metaphysis (distal to coronoid process) with lateral dislocation of the radial head; and type IV, fracture of the proximal or middle third of the ulna and radius with dislocation of the radial head in any direction [8]. Additionally, Jupiter in 1991 subclassified the type II lesions. According to this subclassification: type IIa, an ulna fracture involving the distal end of the olecranon and the coronoid process; type IIb, a metaphyseal-diaphyseal fracture, distal to the coronoid process; type llc, a diaphyseal fracture of the ulna; and type IId, a fracture of the ulna halfway through the bone [9].

Monteggia fractures are mainly associated with falls on an outstretched hand with forced pronation [10]. The decision on operative or non-operative treatment is based on the injury pattern and the patient’s age. Most pediatric fracture patterns can be managed non-operatively with closed reduction and long-arm casting, as long as reduction in the radial head is confirmed, while most adult fractures call for open reduction and internal fixation [11, 12]. More specifically, in children for Bado types I–III fractures, closed reduction in ulna and radial head dislocation and long-arm casting immobilization in 110° of flexion and full supination is preferred. Indications for operative treatment in those cases are: an open fracture, an acute type IV Bado fracture, instability of the radial head or inability to maintain ulnar length [11, 12].

The Galeazzi fracture describes fractures of the distal radial shaft with distal radioulnar joint disruption [13]. This type of injury was first described in 1842 by Cooper. Richardo Galeazzi, an Italian surgeon, reported, 92 years later, a total of 18 cases of such injuries. Since then, this type of fracture became synonymous to his name. The most common mechanism of the Galeazzi fracture is thought to be a fall that causes an axial load placed on a hyperpronated forearm. However, researchers have been unable to reproduce the mechanism of this injury in a laboratory setting [13].

Campbell, in 1941, used the term “fracture of necessity” for the Galeazzi fracture, since it requires surgical treatment. Those injuries are best treated with open reduction in the radius and the distal radioulnar joint [14]. These injuries have been described as very unstable fracture dislocations and therefore are very difficult to treat non-operatively. Causative factors of this instability include: rupture of the triangular fibrocartilage, the complex mechanical association between radius and ulna and the deforming forces of the brachioradialis, pronator quadratus, thumb extensor and abductors. Closed reduction and cast application (non-operative treatment) have led to unsatisfactory results, since they result in persistent or recurrent dislocations of the distal ulna [13–15].

Those kind of injuries combined are extremely rare. Only a few cases of a combined injury in the same extremity have been reported in the international literature, none of which included bipolar fractures [2–5]. In our case, the patient sustained an open bipolar Monteggia, as well as a bipolar Galeazzi injury.

As it was noted in some of the previous similar cases, a fracture involving both forearm bones could be associated with substantial soft tissue injuries, which could disrupt the blood supply [2–5]. In our case, the patient was diagnosed with pseudarthosis, 6 months after initial surgery. This could be attributed to the open fracture, the soft tissue damage, the high degree of initial displacement, the high energy injury, as well as the pattern of the fractures (bipolar). All of which are well-known nonunion predisposing factors [16]. Our case, when compared to the other published cases, had an additional risk factor for nonunion: the bipolar pattern of the injury.

Although not common, these injuries if not treated correctly may lead to deficits, long-term functional impairment and pain to the patient [2–5, 13–15, 17]. The operative treatment in those injuries is essential. In our case, although the patient had to be re-operated, the final functional outcome was satisfactory. The same can be said for most of the other similar cases described, which were also treated operatively.

Forearm fractures often result in occult dislocations in wrist and elbow. Therefore, it is important to keep a keen eye for these injuries and to always examine proximal and distal joints clinically and eventually radiographically for dislocation [17]. A distal radioulnar joint dislocation is not always easy to diagnose. Signs of an occult distal radioulnar joint dislocation that could be looked for in X-ray views are: the loss of radial height and the fracture of the ulnar styloid [14].

Due to the rare occurrence of this combined injury and presence of multiple fractures and dislocations, there is possibility of pitfalls in the management of these cases [14]. Therefore, the treatment of such complex injuries should be performed in the most suitable center, since it depends on the surgeon’s experience and the available resources.
